# Return to sport after arthroscopic rotator cuff repair: epidemiology and prognostic factors in a Swiss multicentre cohort

**DOI:** 10.1136/bjsports-2025-110358

**Published:** 2025-11-20

**Authors:** Madlaina Matter, Laurent Audigé, Thomas Stojanov, Andreas Mueller, Matthias A Zumstein, Annabel Hayoz, ARCR_Pred Study group, Michael Schär, ARCR_Pred Study group, Claudio Rosso, Philipp Moroder, Doruk Akgün, Isabella Weiss, Eduardo Samaniego, Thomas Suter, Sebastian A Müller, Markus Saner, Claudia Haag-Schumacher, Mai LanDao Trong, Carlos Buitrago-Tellez, Julian Hasler, Ulf Riede, Beat Moor, Matthias Biner, Nicolas Gallusser, Christoph Spormann, Britta Hansen, Holger Durchholz, Gregory Cunningham, Alexandre Lädermann, Michael Schär, Rainer Egli, Stephanie Erdbrink, Kate Gerber, Paolo Lombardo, Johannes Weihs, Matthias Flury, Ralph Berther, Christine Ehrmann, Larissa Hübscher, David Schwappach, Karim Eid, Susanne Bensler, Yannick Fritz, Emanuel Benninger, Philemon Grimm, Markus Pisan, Markus Scheibel, Laurent Audigé, Daniela Brune, Marije de Jong, Stefan Diermayr, Marco Etter, Florian Freislederer, Michael Glanzmann, Cécile Grobet, Christian Jung, Fabrizio Moro, Ralph Ringer, Jan Schätz, Hans-Kaspar Schwyzer, Martina Wehrli, Barbara Wirth, Christian Candrian, Filippo Del Grande, Pietro Feltri, Giuseppe Filardo, Francesco Marbach, Florian Schönweger, Bernhard Jost, Michael Badulescu, Stephanie Lüscher, Fabian Napieralski, Lena Öhrström, Martin Olach, Jan Rechsteiner, Jörg Scheler, Christian Spross, Vilijam Zdravkovic, Matthias A Zumstein, Annabel Hayoz, Julia Müller-Lebschi, Karl Wieser, Paul Borbas, Samy Bouaicha, Roland Camenzind, Sabrina Catanzaro, Christian Gerber, Florian Grubhofer, Anita Hasler, Bettina Hochreiter, Roy Marcus, Farah Selman, Reto Sutter, Sabine Wyss, Christian Appenzeller-Herzog, Andreas MarcMüller, Soheila Aghlmandi, Cornelia Baum, Franziska Eckers, Kushtrim Grezda, Simone Hatz, Sabina Hunziker, Thomas Stojanov, Mohy Taha, Giorgio Tamborrini-Schütz, Ilona Ahlborn, Christopher Child, Aleksis Doert, Sebastian Ebert, David Endell, Nikitas Gkikopoulos, Abed Kourhani, Philipp Kriechling, Lucca Lacheta, Daniel Langthaler, Richard Niehaus, Raffaela Nobs, Frederick Schuster, Kathi Thiele, Béatrice Weber

**Affiliations:** 1 Orthopaedic Surgery and Traumatology, University Hospital Basel, Basel, Switzerland; 2 Surgical Outcome Research Center, University Hospital Basel, Basel, Switzerland; 3 Research and Development Department, Shoulder and Elbow surgery, Schulthess Klinik, Zürich, Switzerland; 4 University of Bern Faculty of Medicine, Bern, Switzerland; 5 Sonnenhof Clinic Orthopaedic Department, Bern, BE, Switzerland; 6 Department of Orthopaedic Surgery and Traumatology, Inselspital Bern,University of Bern, Bern, Switzerland

**Keywords:** Sports, Rotator cuff, Epidemiology

## Abstract

**Objectives:**

To investigate patients’ sports activities prior to and after arthroscopic rotator cuff repair (ARCR) and to better understand the relationship between patient, injury and sport-specific factors and return to sport (RTS) after ARCR.

**Methods:**

As a part of the ARCR_Pred multicentre cohort study, patients from 19 centres undergoing primary ARCR for partial or complete rotator cuff tears between June 2020 and November 2021 were prospectively enrolled. Only patients who participated in sports prior to the injury were included. Injury characteristics, sports activity, sociodemographic, psychological and rehabilitation-specific factors, including the ability to return to any sport, were recorded preoperatively and postoperatively at 6 weeks, 6, 12 and 24 months. Prognostic factors for full RTS were identified using univariable and multivariable logistic regression analysis.

**Results:**

Of the 725 eligible patients, 37.2% were female, and the mean age was 57.7 years. Among all eligible patients, 57.4% achieved full RTS at 24 months, and 43.8% returned to their primary preinjury sport. Delayed initiation of passive mobilisation (risk ratio (RR) 0.94 (95% CI 0.89 to 0.99), p=0.017) was associated with incomplete RTS in univariable analysis. In multivariable analysis, favourable prognostic factors of full RTS included traumatic injury aetiology (RR 1.21 (95% CI 1.07 to 1.37), p=0.002), high motivation to RTS at baseline (RR 1.18 (95% CI 1.05 to 1.33), p=0.005), higher sports activity levels at 6 months (RR 1.04 (95% CI 1.01 to 1.07), p=0.002) and a low depression score at 12 months (RR 0.97 (95% CI 0.95 to 0.98), p<0.001).

**Conclusion:**

Over half of ARCR patients reach full RTS within 24 months, with traumatic injuries, high motivation and higher sports activity at baseline having a more favourable prognosis. Our findings inform individualised postoperative rehabilitation and counselling regarding RTS following ARCR.

**WHAT ARE THE FINDINGS?**

- This large multicentre study provides detailed epidemiological data on RTS after ARCR, identifying factors such as traumatic injury, early passive mobilisation, initial motivation, early physical activity and low depression levels as positive predictors of RTS.

**WHAT THIS STUDY ADDS**

- This large multicentre study provides detailed epidemiological data on RTS after ARCR, identifying factors such as traumatic injury, early passive mobilisation, initial motivation, early physical activity and low depression levels as positive predictors of RTS.

**HOW MIGHT IT IMPACT ON CLINICAL PRACTICE IN THE FUTURE?**

- Our findings support the development of tailored rehabilitation strategies that incorporate psychological, activity and injury-related factors to optimise RTS outcomes.
- Future studies should aim to test these tailored rehabilitation strategies on RTS outcomes.

## Introduction

Return to sport (RTS) is a key expectation for patients undergoing elective arthroscopic rotator cuff repair (ARCR).^1^ Various factors such as a higher preoperative sports activity level,^2^ fear of reinjury,^3^ participation in overhead and contact sports,^4^ or specific sports like baseball^5^ have been reported to impede a successful return to the patient’s previous sports activity level. RTS is a multifactorial process in which an athletically active patient advances from general rehabilitation activities to sport-specific movements following an injury.^6^ This progression depends on variables including training intensity, playing position, sport-specific biomechanics^7^ and psychological health and motivation.^8^ Notably, there is no consensus definition of the term RTS in the literature,^9^ with most studies relying on the patient’s subjective perception, primarily assessed using questionnaires.^10–14^

Previous studies analysing the outcome of RTS have focused on a narrow subgroup of patients (eg, only female tennis players,^15^ swimmers,^16^ partial tears,^17^ patients under 45^18^ or over 70^19^ years of age) and are therefore not generalisable. To our knowledge, limited epidemiological data and sports activity levels in a large population of patients with rotator cuff tear (RCT) have been described to date. In 2020, a Swiss-wide multicentre study for evaluation and prediction of core outcomes in ARCR (ARCR_Pred) was initiated.^20^ This study included sports activity levels. Grouping patients based on their RTS outcomes enables the assessment of not only functional recovery, but also outcomes within the complex context of sports participation. This approach could help identify patients who may face greater challenges in returning to their original performance level, allowing for individualised recommendations for those needing additional support during rehabilitation.

The aims of this study were, first, to describe the sports activity levels and epidemiological data of patients undergoing arthroscopic surgical treatment for partial-thickness or full-thickness RCT; second, to determine their return-to-sport rates and to investigate prognostic factors for full RTS. We hypothesised that (1) patients who participate in overhead sports will have more difficulties in returning to the same sport and (2) patients who are more active prior to surgery will more likely RTS.

## Methods

Items included in the present manuscript are reported according to the Strengthening the Reporting of Observational Studies in Epidemiology^21^ guidelines for cohort studies. No patient or public member was involved in the study design.

### Equity, diversity and inclusion statement

Our study included a diverse cohort of patients who underwent ARCR, with no exclusion criteria in terms of age, gender or socioeconomic background. The author group comprised two women and four men with different professional backgrounds and levels of training. All surgeries were performed in clinics in Switzerland or Germany (ie, in wealthier countries), which is why patients from regions with limited access to medical care may not be represented. The influence of age, gender and body mass index on RTS is discussed. However, we did not consider ethnicity, race or socioeconomic status in our analysis.

### ARCR_Pred study setting

Between June 2020 and November 2021, 973 patients undergoing primary ARCR were enrolled across 18 Swiss and 1 German orthopaedic centres and follow-up assessments were conducted up to 24 months postoperatively.^22^ Functional and structural outcomes, as well as patient-reported outcome measures and adverse events (AEs), were recorded. A detailed overview of all measures taken is provided in online esupplemental table 1.

10.1136/bjsports-2025-110358.supp1 Supplementary data

**Table 1:** Baseline characteristics

Parameter	Non-overhead (n=428)		Overhead (n=278)				
	N (%)	Mean (SD)	N (%)	Mean (SD)	Standardised mean difference	Mean difference (95% CI)	P value
Age at surgery (years)		59 (9)		56 (10)	0.283	−3 (−4 to −1)	<0.001
Sex							
Female Male	149 (35) 279 (65)		116 (42) 162 (58)		0.143		0.064
BMI		27 (4)		26 (5)	0.061	−0 (−1 to 0)	0.427
ASA classification							
I II III	186 (43) 216 (50) 26 (6)		142 (51) 121 (44) 15 (5)		0.153		0.139
Surgery on the dominant arm							
No Yes	121 (28) 307 (72)		80 (29) 198 (71)		0.011		0.884
Tear aetiology							
Degenerative Traumatic	198 (46) 230 (54)		124 (45) 154 (55)		0.033		0.666
Tear severity (Gerber *et al*)							
Partial tear Single full tear Two or three tendons Massive tear	64 (15) 108 (25) 71 (17) 185 (43)		40 (14) 81 (29) 39 (14) 118 (42)		0.101		0.631
Pain level NRS (0–10)		5.4 (2.3)		5.1 (2.2)	0.149	−0.3 (−0.7 to 0)	0.054
Sport frequency							
Less than once a week Once a week Two times a week or more	64 (15) 93 (22) 271 (63)		19 (7) 62 (22) 197 (71)		0.265		0.004
Weekly hours of sport		4 (2)		4 (2)	0.191	0 (0 to 1)	0.014
Motivation to return to sport after surgery		10 (1)		10 (1)	0.030	−0 (−0 to −0)	0.695
Confidence to resume sport at 100% of the capacity before the injury		9 (2)		9 (2)	0.153		0.046
PROMIS depression T score		51 (8)		51 (8)	0.002	0 (−1 to 1)	0.979
PROMIS anxiety T score		49 (9)		49 (8)	0.022	−0 (−1 to 1)	0.779

A high degree of similarity between groups was observed across sociodemographic, injury-related and psychological factors.

ASA classification, American Society of Anaesthesiologists Physical Status classification system; BMI, body mass index; NRS, Numerical Rating Scale; PROMIS, Patient Reported Outcomes Measurement Information System.

Baseline documentation included patient demographics, psychological and clinical factors, RCT integrity (ultrasound examination at 12 months by radiologists with musculoskeletal specialty training, experienced rheumatologists or orthopaedic surgeons), concomitant local findings, assessment of the tear aetiology and RCT severity (partial tear | single full-thickness tear | involvement of two or three tendons (one full thickness, the rest partial) | massive tear) by the treating surgeon, surgical details and postoperative management factors. At 6 weeks, the duration of immobilisation, timing of passive and active mobilisation, and type of physiotherapy were recorded. Clinical follow-up at baseline, 6 and 12 months postoperatively included assessment of shoulder pain (patient’s highest pain level experienced in the affected shoulder during ordinary activities within the last 24 hours, documented on a Numerical Rating Scale (NRS) from 0 to 10, maximum pain), range of motion (ROM) (active shoulder flexion, abduction, external rotation at 0° abduction, internal rotation with the Apley’s test) and strength (mean strength in 90° abduction, measured in kg with a handheld dynamometer). Patient-reported questionnaires at baseline, 6, 12 and 24 months postoperatively assessed functional scores, anxiety and depression scores, and participation in sports.

All patients enrolled in the ARCR_Pred study were eligible for inclusion without further exclusion criteria. Patients with previous surgeries, a history of ipsilateral fractures or dislocations of the affected shoulder (n=19) were not excluded.^22^ However, for the assessment of RTS, only patients who reported participation in sport activities at baseline were included.

### Sports activities and overhead classification

At baseline, patients indicated via questionnaire whether they regularly participated in sport activities prior to their RCT. At 6, 12 and 24 months postoperatively, the patients were asked whether they were currently participating in sports. The response options were never, less than once a week, once a week or two times a week or more. Patients also indicated the number of hours spent per week on sports activities. Participants could specify up to three different sports (the most, second most and third most important) that they were currently engaged in. Sports could be selected from a predefined drop-down list with 44 sports, based on the classification system of the Swiss Office of Statistics for the documentation of sports.^23^

Motivation and confidence in RTS at baseline were assessed using a NRS from 0 (not at all) to 10 (fully). The same scale was used to assess motivation to participate in sports at 6, 12 and 24 months. All reported sports were classified as either overhead (eg, swimming, tennis, gymnastics) or non-overhead (eg, cycling, jogging, hiking) based on the most common type of movement, following consensus among the authors. If at least one of the sports specified by the patient at baseline was categorised as an overhead sport, the patient was classified as an overhead-sport patient.

### Return to sport

RTS was defined based on a self-reported assessment provided in the patient questionnaire. At 12 and 24 months postoperatively, patients reported on a NRS from 0 to 10 how close they felt to returning to 100% of their original sport performance. Responses were dichotomised into ‘no full return’ (NRS values 0–8) and ‘full return’ (NRS values 9–10). If patients reported participating in sports at baseline but were not engaging in sports activities at 12 or 24 months, patients were classified as ‘no full return’.

RTS was primarily defined as a composite outcome based on the 24-month questionnaire value. If the 24-month questionnaire was not available (n=37), the value of the 12-month questionnaire was used. In addition, we documented whether patients were able to return to the same type of sport as reported at baseline.

### Studied risk factors

In addition to sports activity parameters (frequency, quantity, sport, motivation and confidence) at baseline and at 6 months, a set of potential risk factors available in the ARCR_Pred study was investigated for associations with RTS. These included patient sociodemographic characteristics, surgical and injury-related factors, parameters related to the rehabilitation procedure, preoperative and postoperative scores up to 12 months and complications. Scores included: pain level, the Constant-Murley Score,^24^ abduction strength, a Shoulder Stiffness Scale (SSS) and psychological Patient Reported Outcomes Measurement Information System (PROMIS) depression and anxiety T-scores (calculated with the HealthMeasures PROMIS Scoring Manuals).^25^ ^26^ The SSS is a monitoring tool for postoperative shoulder stiffness and ranges from 0 to 10 points (maximum stiffness). It is calculated from the parameters pain (sss1: 0–3 points), subjective ROM limitation in daily activities (sss2: 0–3 points), as well as side-to-side difference in passive external rotation in adduction and glenohumeral abduction (sss3: 0–4 points). Complications included events involving the operated shoulder, such as stiffness events within 6 months requiring treatment, and ipsilateral AEs within 12 and 24 months.

### Data management and statistical analysis methods

Study data were managed using the REDCap Electronic Data Capture system^27^ and exported for variable transformation (including score calculations) and statistical analysis using Stata V.17 (StataCorp LP, College Station, Texas, USA).

#### Sports activities and baseline characteristics

Demographic characteristics, health status, intraoperative findings, sports activity levels and clinical shoulder parameters at baseline were analysed using descriptive statistics (mean and SD; frequency and percentages, as appropriate). Overhead and non-overhead sports patient groups were compared in terms of baseline characteristics using standardised mean differences (Std. Diff.).

#### Risk factors for RTS

An initial list of 36 candidate predictors for RTS was identified using expert-based knowledge from clinicians involved as a coauthor in the present study (AM, MAZ and MS). A univariable Poisson regression analysis was conducted to investigate the association (risk ratio (RR) and corresponding 95% CIs) between the aforementioned factors and the outcome of ‘full RTS’.^28^ For continuous and ordinal variables, we assessed non-linearity trends in univariable models using splines with one knot. The percentage of missing values for specific variables ranged from 0 to 14.8%. The proportion of patients with at least one missing predictor was 10%. Assuming that these missing values were missing at random, multiple imputation was performed using chained equations^29^ with 20 data sets. Missing values were imputed for risk factors only, not for the outcome ‘full RTS’.

To examine the associations between multiple factors simultaneously and the RTS outcome, a multivariable Poisson regression analysis was performed. Given a fixed sample size (n=725), outcome prevalence of 42.6% and an anticipated c-statistic of 0.7 based on previous findings,^30^ we decided to restrict the model to a maximum of 10 factors (using the Stata package *pmsampsize,* following recommendations by Riley *et al* 31). The relationship between any factor pairs was analysed using correlation coefficient for continuous variables, χ² test for categorical variables (after checking statistical assumptions) and Spearman’s rank correlation coefficient for comparison of a continuous with a categorical variable.^32^ When two variables described similar constructs, variables that would lead to less loss of information (eg, number of hours of sport was preferred over the categorised sports frequency) were kept. Similarly, when correlation was observed between two factors (correlation coefficient >0.3,^32^ p value of χ² test <0.05,^33^ Spearman’s rank correlation coefficient >0.3),^32^ only one of the variables was retained prior to the variable selection procedures. A final exploratory RTS model was identified using backward elimination as the variable selection strategy with an alpha level of 0.157.^34^ Variable selection was performed on the complete case data. Model performance was assessed using Akaike’s Information Criterion (AIC),^35^ the generalised index of concordance (c-index)^36^ and Pearson goodness-of-fit.^36^ In the last step, when there were signs of non-linearity between continuous and/or ordinal variables and the outcome, we assessed whether non-linearity terms contributed to an improvement of model performance.^35 36^

## Results

### Patient cohort selection

**Figure 1:**
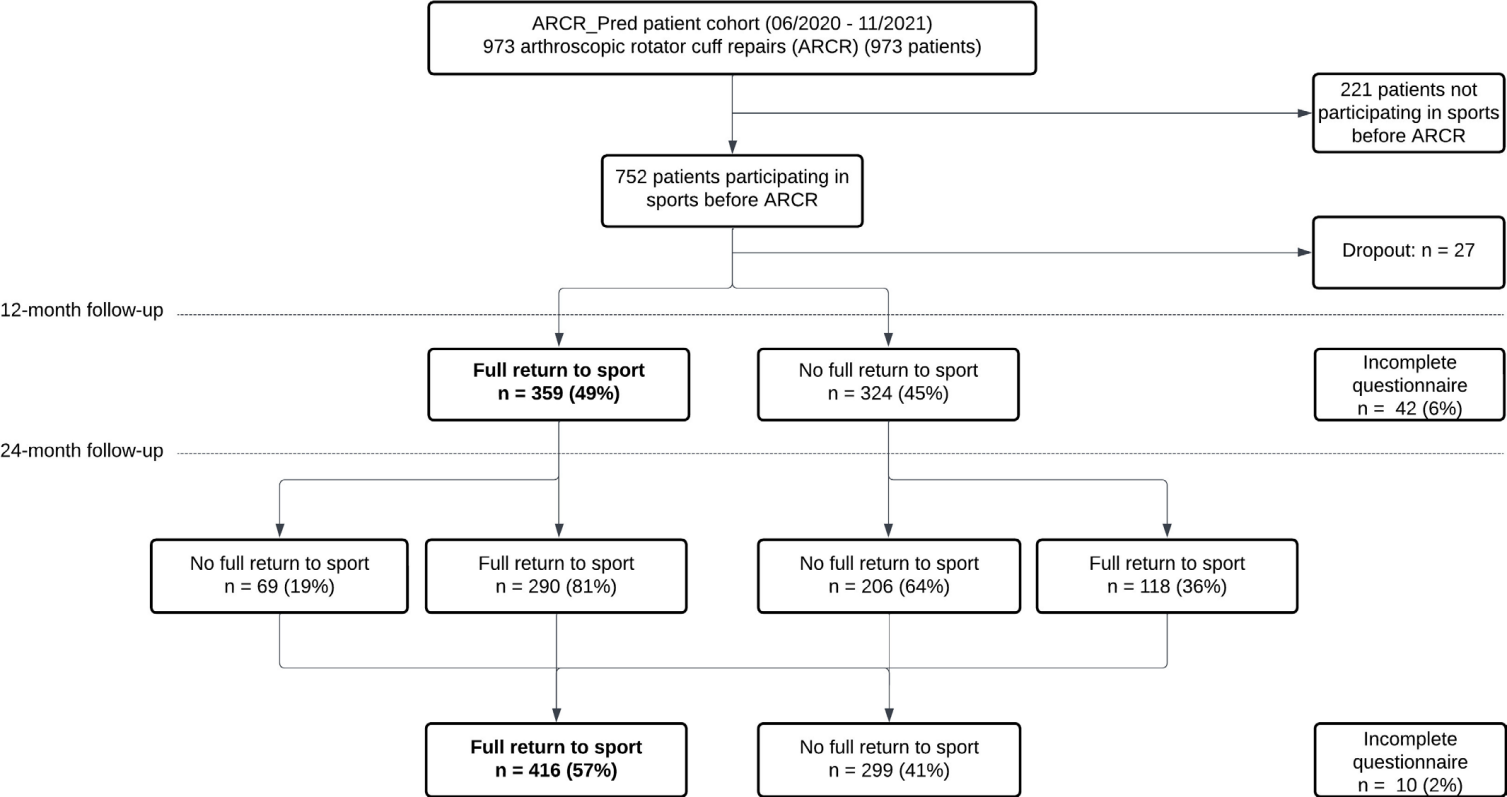
Patient flow chart and follow-up. This figure presents patient selection, follow-up and RTS rates. RTS, return to sport.

Of the 973 patients enrolled in the ARCR_Pred study, 752 (77.3%) were participating in sport at baseline. Of these, 27 patients were lost to follow-up before the 12-month follow-up, leaving 725 patients (74.5%) available for analysis (figure 1). Among these, 37.2% were female, and the mean age was 57.7 years (range: 21–84 years).

The severity of the RCT was classified intraoperatively as follows: 15.0% had a partial tear, 26.2% a single full-thickness tear, 15.5% involvement of two or three tendons (one full thickness, the rest partial) and 43.3% a massive tear. The tear was associated with a traumatic event in 54.2% of cases. The dominant shoulder was injured in 71.5% of ARCR cases.

### Sport participation and comparison of baseline characteristics

Overall, the three most frequently reported sports were cycling (n=231), jogging/running (n=189) and hiking/mountaineering (n=173) (online esupplemental table 2). Of the cohort, 278 (38.3%) were classified as overhead-sport patients and 428 (59.0%) as non-overhead-sport patients (2.6% of patients did not specify their sport). The most practised overhead sports were swimming (n=114), weight training (n=65) and tennis (n=49).

10.1136/bjsports-2025-110358.supp2 Supplementary data

**Figure 2:**
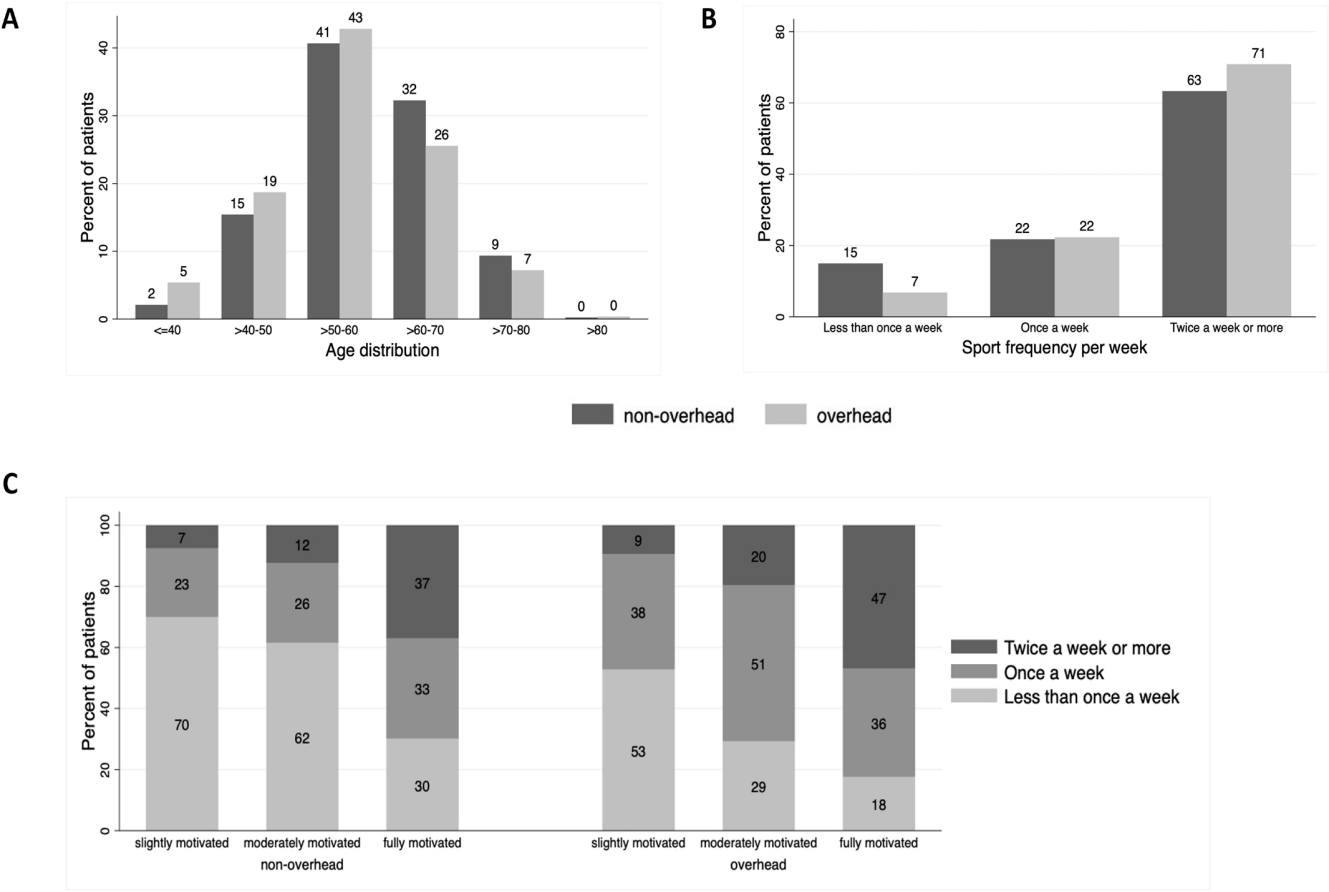
Distribution of baseline characteristics. (A and B) Show the distribution of age (A) and sport frequency per week (B) among patients participating in overhead versus non-overhead sports. (C) Compares sport frequency per week with motivation to return to sport, grouped by overhead versus non-overhead-sport participation.

Patients in the non-overhead-sport group were older on average (59 vs 56 years, Std. Diff.=0.283) and exercised fewer times per week than those in the overhead-sport group (figure 2A,B). Tear aetiology (Std. Diff.=0.033) and severity (Std. Diff.=0.101) were similar between the groups. The preoperative motivation to RTS (mean 10 (SD=2), Std. Diff.=0.030) and confidence to resume sport at 100% of their original capacity (mean 9 (SD=2), Std. Diff=1.53) was high in both groups. Patients who exercised at least two times a week prior to surgery were more motivated to RTS than those who exercised less than once a week (Std. Diff.=0.716, figure 2C). A detailed overview of all baseline characteristics is provided in table 1.

### Return to sport

**Figure 3:**
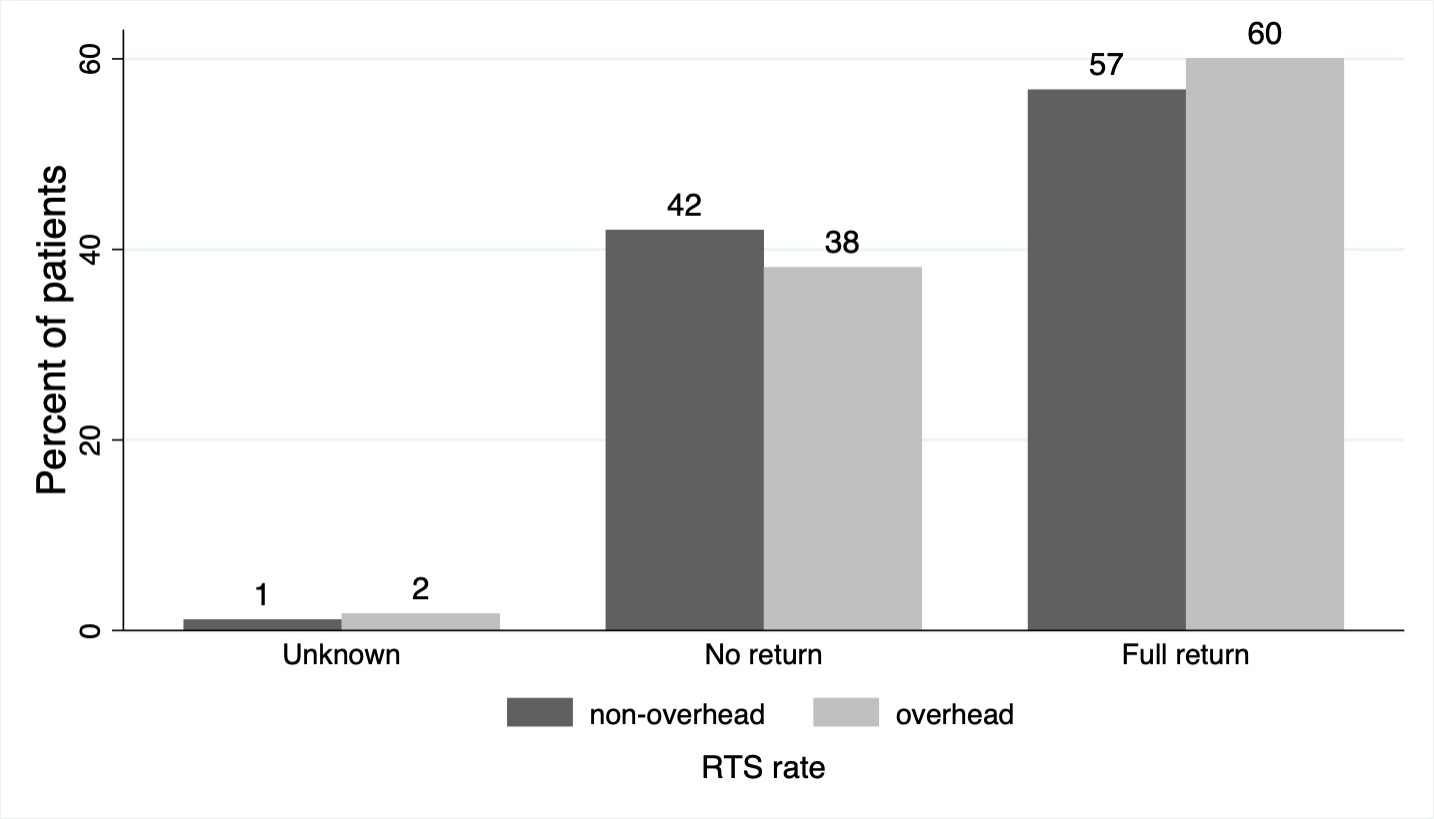
RTS rates per sport group. This figure shows a comparison of RTS rates between patients participating in overhead and non-overhead sports. RTS, return to sport.

At 12 months, 49.5% (359/725) of patients had already reached full RTS; by 24 months, this proportion increased to 57.4% (416/725). Overall, 60.1% (167/278) of patients in the overhead-sport group achieved full RTS within 24 months, compared with 56.8% (243/428) in the non-overhead-sport group (figure 3).

Among patients who achieved full RTS within 24 months, 90.1% (375/416) returned to at least one sport they had practised before the injury.

**Figure 4:**
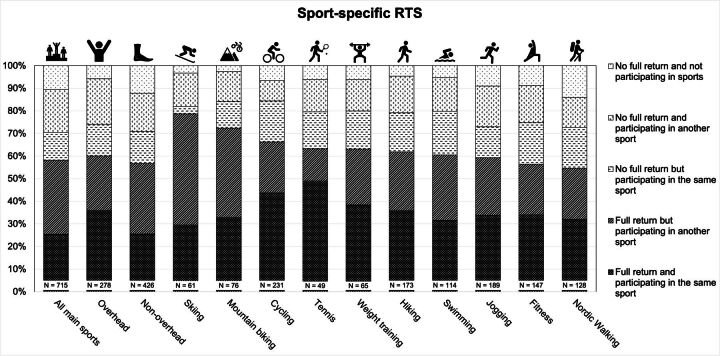
Sport-specific RTS rates. This figure shows RTS rates for the 10 most practised sports. It also indicates whether patients were able to return to the same sport as they reported at baseline. As patients could list up to three different sports, some may be included in multiple categories. RTS, return to sport.

A full RTS to the same main sport was observed in 43.8% (182/416). Among overhead-sport patients, 36.0% (100/278) returned to their original overhead sport. Among non-overhead sport patients, 25.6% (50/123) returned to their original non-overhead sport. A detailed overview of all individual sports and their RTS outcomes is presented in figure 4 and online esupplemental table 2.

### Risk factors for RTS

The analysis of 36 risk factors for RTS is summarised in online esupplemental table 3. At the univariable analysis level, several sociodemographic, surgical and injury-related factors showed a significant association with full RTS (p<0.05). These included, for example, dominance of the operated side (RR 0.87 (95% CI 0.76 to 0.99); p=0.031), age at surgery (RR 1.01 (95% CI 1.01 to 1.02); p<0.001) and traumatic injury (RR 1.20 (95% CI 1.06 to 1.37); p=0.005).

10.1136/bjsports-2025-110358.supp3 Supplementary data

The time of onset of passive movements in weeks (RR 0.94 (95% CI 0.89 to 0.99); p=0.017), a higher baseline level of sports activity, all preoperative and postoperative scores and psychological factors at almost all follow-up points, particularly the motivation to RTS at baseline (RR 1.29, (95% CI 1.15 to 1.44), p<0.001), were significantly associated with full RTS. Practising an overhead sport (RR 1.06 (95% CI 0.94 to 1.21); p=0.325) was not significantly associated with RTS outcome.

**Figure 5:**
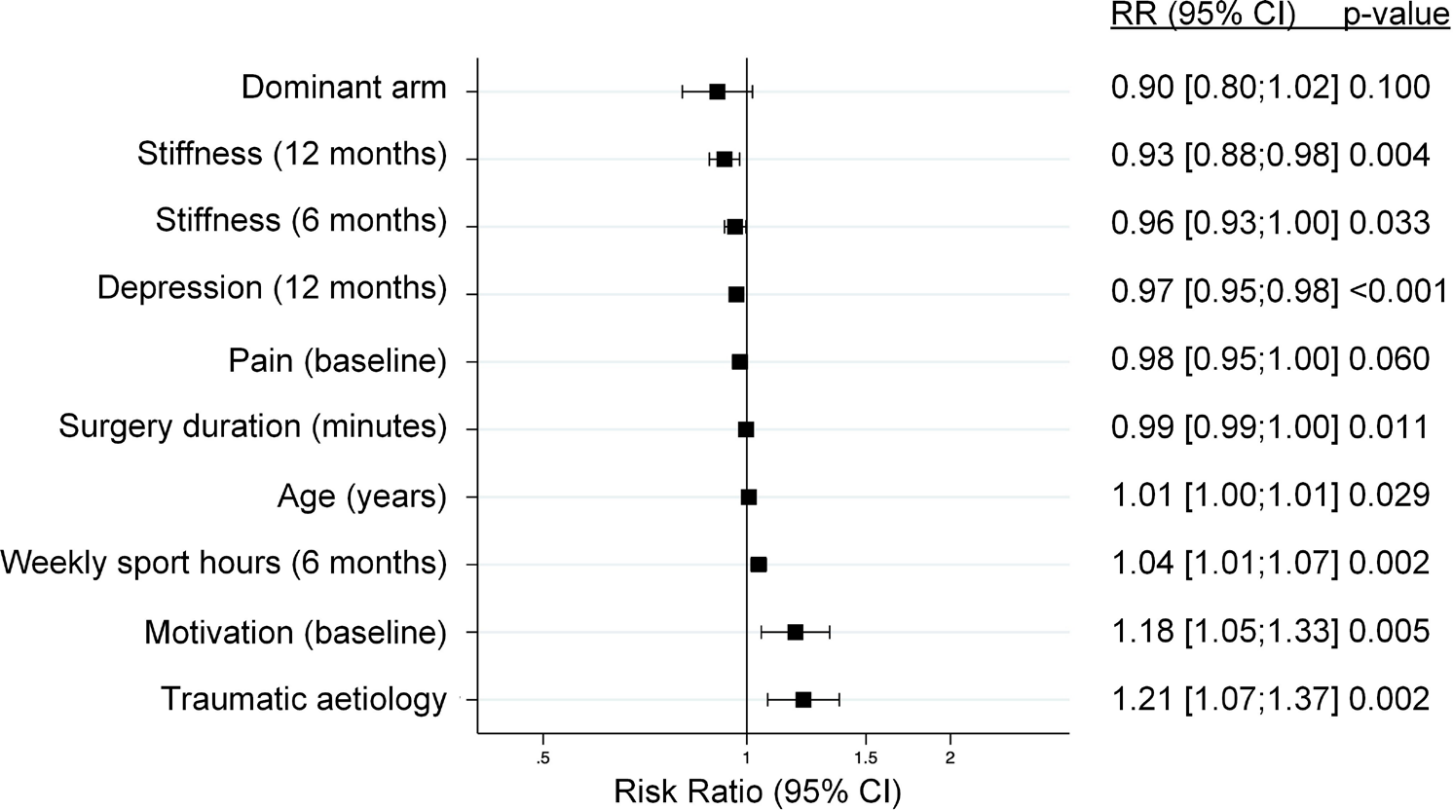
Multivariable logistic regression model. This figure shows the forest plot of the multivariable model including 10 factors. Squares represent the RR, and horizontal lines indicate 95% CIs of the specific factor. RR, risk ratio.

From the initial list of 36 candidate predictors, 8 were dropped because of high correlation and redundant information. The final model resulting from the backward elimination procedure was composed of 10 factors (figure 5, online esupplemental table 4a). Inclusion of non-linear spline terms did not improve the performance of the final multivariable model. The model performance was AIC=1130.708, Pearson goodness-of-fit = 244.082 and c-index=0.762 (online esupplemental table 4b).

10.1136/bjsports-2025-110358.supp4 Supplementary data

## Discussion

We investigated the ability of patients in Switzerland to RTS after undergoing an ARCR. Overall, 57.4% were able to RTS following ARCR. While RTS rates did not differ between overhead and non-overhead sports, we observed variation at the level of individual sports. Patients were more likely to RTS in sports such as tennis and cycling, and less likely in swimming and skiing. We also only noted minor differences in baseline characteristics between patients participating in overhead versus non-overhead sports. Therefore, differences in RTS between these two sports groups may not be biased by confounding baseline conditions, aside from age and level of sports activity.

The baseline distributions of sports participation in our investigation were similar to those among the Swiss population in 2020 reported by the Swiss Federal Office for Sport.^23^ A direct comparison of RTS rates with the literature is challenging because the studies differ greatly in terms of patient selection, injury characteristics and definitions of RTS. Yet, meta-analyses have reported higher rates of return to the same or better level (65.9%^2^ and 70.2%,^37^ respectively). In our study, participation in a specific sport group such as overhead sports had no clear influence on RTS rates. Therefore, our first hypothesis was rejected. This finding may appear unexpected, given the high precision and function required of the shoulder in overhead sports,^38^ and contrasts with previous studies^2 37^ that identified participation in overhead sports as a potential negative predictor of RTS. A large part (90%) of patients returned to at least one sport that they had practised prior to injury, but only 25% returned to their primary sport. High RTS rates were achieved by patients participating in golf, tennis or cycling, consistent with findings from previous studies.^39 40^ Regardless of sport type, patients who exercised more frequently prior to surgery were associated with higher RTS rates, confirming our second hypothesis.

In the univariable analysis, early passive mobilisation was significantly associated with full RTS. The start of passive mobilisation is a balancing act between early functional activity to prevent stiffness and enhance shoulder mobility and impairing tendon healing.^41^ Its absence from the multivariable model may be due to the multiple interrelationships among the various risk factors assessed.^42^ The SSS itself showed a significant correlation with complete RTS both in the univariable analysis and multivariable model, suggesting its prognostic value in orthopaedic shoulder surgery. A shorter duration of surgery, also found to be a predictor in other studies,^43 44^ appears to serve as a proxy for disease complexity. This may be due to factors such as the surgeon’s experience or the complexity of the rupture.^45 46^

All psychological factors assessed in our study had a significant impact on RTS similar to previous studies.^47 48^ This emphasises the importance of the patient’s attitude towards surgery and mindset in the subsequent recovery period and may encourage the integration of mental health considerations into preoperative and postoperative assessments. However, high motivation may also carry the risk of premature return to training, potentially resulting in relapse or a new injury.

The strengths of this study include the large sample size with a total of 725 rotator cuff repairs and the minimal exclusion criteria, limited only to the absence of sports activity at baseline. As a result, findings can be generalised to physically active patients with RCT. Additionally, the 24-month follow-up period provides an overview of a longer period during which functional improvements or also deteriorations can be observed.

Our definition of RTS was based on the patient’s subjective assessment of their ability to participate in sport. Therefore, it may also have been influenced by individual levels of motivation or psychological burden. Patients with lower motivation or more pronounced depressive symptoms may have perceived their condition less positively.^49^ In addition, RTS was assessed as a general outcome, limiting the precision of our analysis for specific sport types. This assessment may also explain the relatively low rate (43.8%) of full return to the same main sport: a change in ranking (eg, from first to second priority) may not be indicative of a poor outcome but may rather reflect changes in personal preferences. Also, we did not collect data on the exact time point at which patients returned to sport, which limits time-based outcome analyses. We acknowledge that measurements like, for example, strength in other motions than abduction are lacking. These additional strength measures were not documented in our database. Some potential confounding factors, such as surgeon’s experience, differences in rehabilitation adherence or structural tendon healing, were not considered in our analysis.

We developed an exploratory model with 10 predictors aiming at identifying factors associated with full RTS after ARCR.^36^ The model showed a sound predictive accuracy compared with other recent orthopaedic models.^30 50^ However, we must acknowledge that the selection of the 10 predictors in this model was data-driven and therefore prone to testimation bias^35^ and could lead to overfitting. If the aim was to develop and validate a proper prediction model for RTS, there would remain room to improve model specification and performance by making use of expert knowledge (eg, using Delphi surveys) and/or advanced variable selection procedure methods.^51^

## Conclusion

This study examined the relationship between epidemiological factors and sports activity in physically active patients who underwent ARCR. Over half of ARCR patients achieved full RTS, with nearly all returning to a sport they had already practised before surgery. Traumatic injury aetiology, high motivation to RTS, higher sports activity levels and a low depression score were associated with full RTS within 2 years after ARCR. Based on these findings, a multivariable model for full RTS after ARCR was established, contributing to the evolving field of prognostic modelling for ARCR outcomes.

## Data Availability

Data are available upon reasonable request. Following a minimum embargo of two years after study completion in September 2024, metadata describing the type, size and content of the data set will be made publicly available alongside the study protocol via the open-access repository Zenodo (https://zenodo.org/). Requests for full data set access may be submitted to the Data Access Committee of the Medical Faculty of the University of Basel (MF-DAC) via email (med-dac@unibas.ch). Access will be granted upon independent review and confirmation that ethical, legal and scientific requirements are met.
