# Supplementary material for: Return to sport after arthroscopic rotator cuff repair: epidemiology and prognostic factors in a Swiss multicentre cohort
Source: Br J Sports Med. 2025 Nov 20;60(2):116–24. doi: 10.1136/bjsports-2025-110358 (PMC12916472; doi:10.1136/bjsports-2025-110358)
Supplement: Supplementary data [file bjsports-60-2-s001.pdf]

eSupplement Table 1: **Variable list**

	Baseline	6 weeks	6 months	12 months	24 months	Variable explanation	Units
**Baseline documentation**							
Sex	x					Sex	1, Male | 0, Female
Age (years)	x					Age at surgery	Numeric
Body mass index (BMI) (kg/m^2)	x					Weight (kg) / (Height (m))^2	Numeric
ASA classification	x					ASA classification	1, ASA 1: Healthy person | 2, ASA 2: Patient with mild systemic disease | 3, ASA 3: Patient with severe systemic disease | 4, ASA 4: Patient with severe systemic disease that is a constant threat to life
Current smoker	x					Smoking status	1, Yes | 0, No
Dominant side operated	x						1, Dominant | 0, Non-dominant
Assessment of tear aetiology	x					Surgeon's judgement as to the cause of the shoulder complaints / rotator cuff tear	1, Purely degenerative | 2, More degenerative than traumatic | 3, More traumatic than degenerative | 4, Purely traumatic
**Operation form and postoperative management**							
Tear severity (Gerber classification)	x					Intraoperative diagnosis	1, Partial tear | 2, Single full tear | 3, Two or three tendons (only one full) | 4, Massive tear (Gerber et al)
Subscapularis tear	x					Intraoperative diagnosis	1, Yes | 0, No
Supraspinatus tear	x					Intraoperative diagnosis	1, Yes | 0, No
Infraspinatus tear	x					Intraoperative diagnosis	1, Yes | 0, No
Operation duration (minutes)	x						Numeric
Concomitant injuries	x					Concomitant injuries/observations, intraoperative diagnosis	0, None | 1, Superior Labral tear from Anterior to Posterior (SLAP) lesion | 2, Humeral Avulsion Glenohumeral Ligament (HAGL) lesion | 3, Bankart lesion | 4, Humerus cartilage lesion (ICRS grading ≥3 : defect >50% of cartilage depth) | 5, Glenoid cartilage lesion (ICRS grading ≥3 : defect >50% of cartilage depth) | 6, Acromioclavicular joint degeneration | 9, Other concomitant injuries / observations
Concomitant interventions	x					Concomitant operative treatment/interventions	1, Acromioclavicular joint resection | 2, Acromioplasty | 3, Capsulotomy | 4, Platelet concentrates | 5, Patch (graft) augmentation | 6, Tendon transfer → exclusion | 9, Other

							concomitant treatment(s)
Duration of immobilization (weeks)		x					0, No immobilization | 1, 1 week | 2, 2 weeks | 3, 3 weeks | 4, 4 weeks | 5, 5 weeks | 6, 6 weeks | 7, 7 weeks | 8, 8 weeks
Start of passive movements (weeks)		x					1, At day 1 | 2, After 1 week | 3, After 2 weeks | 4, After 3 weeks | 5, After 4 weeks (1 month) | 6, After 5 weeks | 7, After 6 weeks | 8, After 7 weeks | 9, After 8 weeks (2 months)
Start of active movements (weeks)		x					1, At day 1 | 2, After 1 week | 3, After 2 weeks | 4, After 3 weeks | 5, After 4 weeks (1 month) | 6, After 5 weeks | 7, After 6 weeks | 8, After 7 weeks | 9, After 8 weeks (2 months) | 10, After 9 weeks | 11, After 10 weeks | 12, After 11 weeks | 13, After 12 weeks (3 months)
Rehabilitation procedure		x				Rehabilitation procedure started	0, None | 1, Physiotherapy | 2, Watertherapy | 3, Ergotherapy | 7, Replacement sport training without arms | 9, Other rehabilitation procedure(s)
Duration of home exercises (weeks)			x	x		Total duration since start of training	0 - 35 weeks
**Clinical assessment (baseline and follow-up)**							
Pain level (NRS 0-10)	x		x	x		Patient's highest pain level experienced in the affected shoulder during ordinary activities within the last 24 hours.	0 (no pain) -10 (maximum pain)
ROM (active flexion, active abduction, external rotation at 0°, internal rotation with the Apley's test)	x		x	x		Flexion active (°), Abduction active (°), External rotation at 0° abduction (active) (°), internal rotation with the Apley's test	Flexion and abduction from 0°-200°;<br>External rotation at 0° from 0-100°;<br>Internal rotation:0, Lateral thigh | 2, Buttock | 4, Lumbosacral junction | 6, Waist (L3) | 8, T12 vertebra | 10, Interscapular T7
Mean strength in 90° abduction (kg)	x		x	x		Mean strength of the affected side in 90° abduction (kg) measured with a handheld dynamometer	Numeric
Shoulder Stiffness Scale	x		x	x		sss1 (pain: 0 to 3 points) + sss2 (subjective ROM limitation in daily activities: 0 to 3 points) + sss3 (side-to-side difference in passive external rotation in adduction and glenohumeral abduction: 0	0-10

						to 4 points)	
Total Constant-Murley Score	x		x	x		Pain (0-15 points) + daily activity (0-20 points) + motion (0-40 points) + strength (0-25 points)	0-100
PROMIS anxiety T-score	x		x	x	x		0-100
PROMIS depression T-score	x		x	x	x		0-100
RCT integrity				x		Ultrasound examination at 12 months by radiologists with musculoskeletal specialty training, experienced rheumatologists, or orthopaedic surgeons	0 No defect | 1 Partial defect | 2 Full-thickness defect
Stiffness within 6 months			x			Stiffness event within 6 months requiring treatment	1, Yes | 0, No
Ipsilateral adverse events			x	x	x	Occurrence of at least one adverse event affecting the ipsilateral arm within 24 months	1, Yes | 0, No
**Patient-reported questionnaires**							
Type of sport (max. 3)	x		x	x	x	What kind of sports do you do? (the most important, the second most important, the third most important)	Dropdown list of 44 different sport types
Overhead sport patient	x		x	x	x	Swimming, Weight training / Bodybuilding, Tennis, Gymnastics, Martial arts / Self defence, Badminton, Climbing / mountaineering, Volleyball / Beach volleyball, Windsurfing / Surfing / Kitesurfing, Handball, Squash, Basketball	1, Yes | 0, No
Non-overhead sport patient	x		x	x	x	All other reported sports	1, Yes | 0, No
Sport frequency per week	x		x	x	x	At baseline: Do you usually do sports; in the follow-up: Are you currently doing sports?	0, Never | 1, Less than once a week | 2, Once a week | 3, Twice a week or more

Hours of sport per week	x		x	x	x	Number of hours per week	1, < 2 hours | 2, 2 | 3, 3 | 4, 4 | 5, 5 | 6, 6 | 7, 7 and more hours
Motivation to do sports again after surgery	x					How motivated are you to do sports again after the operation?	0 (not at all) -10 (fully)
Current motivation to do sports				x	x	How are you currently motivated to do sports?	0 (not at all) -10 (fully)
Confidence to resume sport at 100% of the initial capacity	x					How confident are you that you will be able to resume sport at 100% of your capacity after the operation?	0 (not at all) -10 (fully)
Ability to resume sport at 100% of the initial capacity				x	x	Were you able to resume sport at 100% of the capacity you had before your shoulder injury?	0 (not at all) -10 (fully)

ASA classification = American Society of Anesthesiologists Physical Status classification system; BMI = Body Mass Index; ROM = Range of Motion
