# Supplementary material for: Return to sport after arthroscopic rotator cuff repair: epidemiology and prognostic factors in a Swiss multicentre cohort
Source: Br J Sports Med. 2025 Nov 20;60(2):116–24. doi: 10.1136/bjsports-2025-110358 (PMC12916472; doi:10.1136/bjsports-2025-110358)
Supplement: Supplementary data [file bjsports-60-2-s002.pdf]

eSupplement Table 2: **Sport participation and RTS rates**.

Sport types	Baseline (N)	Full RTS to the same sport N (%)	Full RTS but not to the same sport N (%)	No full RTS but to the same sport N (%)	No full RTS and not to the same sport N (%)	Not doing sport N (%)
**All main sports**	715	182 (25)	234 (33)	88 (12)	136 (19)	75 (10)
**Overhead sport**	278	100 (36)	67 (24)	39 (14)	56 (20)	16 (6)
Swimming	114	36 (32)	33 (29)	22 (19)	17 (15)	6 (5)
Weight training / Bodybuilding	65	25 (38)	16 (25)	11 (17)	9 (14)	4 (6)
Tennis	49	24 (49)	7 (14)	8 (16)	7 (14)	3 (6)
Gymnastics	35	8 (23)	8 (23)	3 (9)	14 (40)	2 (6)
Martial arts / Self-defence	14	3 (21)	2 (14)	1 (7)	6 (43)	2 (14)
Badminton	10	3 (30)	3 (30)	3 (30)	1 (10)	0 (0)
Climbing / mountaineering	9	2 (22)	1 (11)	2 (22)	3 (33)	1 (11)
Volleyball / Beach volleyball	8	5 (63)	1 (13)	1 (13)	1 (13)	0 (0)
Windsurfing / Surfing / Kitesurfing	5	1 (20)	1 (20)	1 (20)	2 (40)	0 (0)
Handball	3	1 (33)	0 (0)	2 (67)	0 (0)	0 (0)
Squash	3	1 (33)	0 (0)	1 (33)	0 (0)	1 (33)
Basketball	2	0 (0)	1 (50)	0 (0)	1 (50)	0 (0)
**Non-overhead sports**	426	109 (26)	133 (31)	60 (14)	72 (17)	52 (12)
Cycling (without mountain biking)	231	101 (44)	52 (23)	42 (18)	21 (9)	15 (6)
Jogging / Running	189	64 (34)	48 (25)	26 (14)	34 (18)	17 (9)
Hiking / Mountaineering	173	62 (36)	45 (26)	30 (17)	28 (16)	8 (5)
Fitness Training / Aerobics	147	50 (34)	33 (22)	27 (18)	24 (16)	13 (9)
Walking / Nordic Walking	128	41 (32)	29 (23)	23 (18)	17 (13)	18 (14)
Mountain biking	76	25 (33)	30 (39)	9 (12)	10 (13)	2 (3)
Skiing (without ski tours)	61	18 (30)	30 (49)	2 (3)	9 (15)	2 (3)
Yoga / Tai Chi / Qi Gong	48	17 (35)	10 (21)	11 (23)	9 (19)	1 (2)
Ski/snowboard tours / Snowshoeing	31	5 (16)	13 (42)	4 (13)	7 (23)	2 (6)
Dancing (incl. jazz dance)	25	11 (44)	7 (28)	3 (12)	4 (16)	0 (0)
Football	21	8 (38)	3 (14)	3 (14)	7 (33)	0 (0)
Golf	20	13 (65)	3 (15)	4 (20)	0 (0)	0 (0)
Aqua-Fitness	19	9 (47)	4 (21)	4 (21)	1 (5)	1 (5)

eSupplement Table 2 (continued)

Cross-country skiing	16	1 (6)	10 (63)	2 (13)	3 (19)	0 (0)
Sailing	11	3 (27)	4 (36)	3 (27)	0 (0)	1 (9)
Horse riding / Equestrian sports	10	4 (40)	2 (20)	1 (10)	2 (20)	1 (10)
Inline Skating / Skating	7	2 (29)	3 (43)	1 (14)	0 (0)	1 (14)
Ice Hockey	6	3 (50)	3 (50)	0 (0)	0 (0)	0 (0)
Rowing	5	2 (40)	2 (40)	1 (20)	0 (0)	0 (0)
Floorball (incl. roller hockey)	4	1 (25)	1 (25)	1 (25)	1 (25)	0 (0)
Table tennis	4	1 (25)	1 (25)	1 (25)	0 (0)	1 (25)
Ice Skating	3	0 (0)	2 (67)	0 (0)	0 (0)	1 (33)
Snowboarding (excl. tours)	3	1 (33)	0 (0)	0 (0)	2 (67)	0 (0)
Diving	2	1 (50)	0 (0)	1 (50)	0 (0)	0 (0)
Athletics	1	0 (0)	1 (100)	0 (0)	0 (0)	0 (0)
Shooting	1	0 (0)	0 (0)	1 (100)	0 (0)	0 (0)

Patients may appear in multiple sports, as up to three sports could be declared. 10 patients did not specify their sport (e.g., other team and game sports, other adventure and experience sports, other endurance sports, or other sports) and are therefore not listed. Same sport refers to returning to the same sport as before the injury. RTS = Return to Sport.
