# Supplementary material for: Return to sport after arthroscopic rotator cuff repair: epidemiology and prognostic factors in a Swiss multicentre cohort
Source: Br J Sports Med. 2025 Nov 20;60(2):116–24. doi: 10.1136/bjsports-2025-110358 (PMC12916472; doi:10.1136/bjsports-2025-110358)
Supplement: Supplementary data [file bjsports-60-2-s003.pdf]

eSupplement Table 3: **Univariable associations of risk factors for RTS**

	Complete case					Multiple imputed data	
	Missing (%)	No full return N (%)	Full return N (%)	RR (95% CI)	p-value	RR (95% CI)	p-value
**Sociodemographic factors (N = 5)**							
Age at surgery (years) [Mean (SD)]	0	56 (10)	59 (9)	1.01 [1.01; 1.02]	<0.001	1.01 [1.01; 1.02]	<0.001
Male Sex	0	181 (61)	265 (64)	1.06 [0.93; 1.21]	0.394	1.03 [0.89; 1.18]	0.677
BMI (kg/m^2) [Mean (SD)]	0	27 (5)	26 (4)	0.98 [0.96; 0.99]	0.003	0.97 [0.96; 0.99]	0.001
ASA classification	0						
I		123 (41)	208 (50)	Ref	Ref	Ref	Ref
II		156 (52)	186 (45)	0.87 [0.76; 0.98]	0.027	0.83 [0.73; 0.95]	0.007
III - IV		20 (7)	22 (5)	0.83 [0.62; 1.13]	0.235	0.82 [0.60; 1.12]	0.215
Current smoker	0	66 (22)	53 (13)	0.73 [0.59; 0.90]	0.004	0.63 [0.50; 0.81]	<0.001
**Surgical and injury-related factors (N = 10)**							
Dominant side operated	0	226 (76)	285 (69)	0.87 [0.76; 0.99]	0.031	0.90 [0.78; 1.03]	0.117
Traumatic aetiology	0	144 (48)	245 (59)	1.20 [1.06; 1.37]	0.005	1.24 [1.08; 1.42]	0.002
Subscapularis tear	0	137 (46)	192 (46)	1.01 [0.89; 1.14]	0.930	1.01 [0.88; 1.15]	0.917
Supraspinatus tear	0	278 (93)	382 (92)	0.94 [0.75; 1.16]	0.554	0.92 [0.73; 1.15]	0.459
Infraspinatus tear	0	133 (44)	166 (40)	0.92 [0.81; 1.05]	0.226	0.89 [0.78; 1.02]	0.100
Tear severity (Gerber classification)	0						
Partial tear		43 (14)	63 (15)	Ref	Ref	Ref	Ref
Single full tear		73 (24)	116 (28)	1.03 [0.85; 1.25]	0.745	1.07 [0.88; 1.31]	0.502
Two or three tendons (only one full)		51 (17)	59 (14)	0.90 [0.71; 1.14]	0.391	0.88 [0.68; 1.13]	0.309
Massive tear		132 (44)	178 (43)	0.97 [0.80; 1.16]	0.714	0.98 [0.81; 1.18]	0.829
Operation duration (minutes) [Mean (SD)]	0	82 (35)	73 (30)	1.00 [0.99; 1.00]	0.001	0.99 [0.99; 1.00]	0.001
Acromioclavicular joint resection	0	28 (9)	25 (6)	0.80 [0.35; 1.08]	0.094	0.84 [0.63; 1.13]	0.246

Acromioplasty	0	167 (56)	273 (66)	1.19 [1.04; 1.37]	0.010	1.18 [1.03; 1.36]	0.020
Capsulotomy	0	16 (5)	19 (5)	0.93 [0.68; 1.27]	0.646	0.98 [0.72; 1.33]	0.889
**Sport activity (N=5)**							
Practicing an overhead sport at baseline	0	106 (35)	167 (40)	1.06 [0.94; 1.21]	0.325	1.07 [0.94; 1.22]	0.326
Sport frequency at baseline	0						
Less than once a week		53 (18)	37 (9)	Ref	Ref	Ref	Ref
Once a week		74 (25)	84 (20)	1.29 [0.97; 1.72]	0.080	1.43 [1.06; 1.96]	0.022
Twice a week or more		172 (58)	295 (71)	1.54 [1.19; 1.99]	0.001	1.66 [1.25; 2.20]	<0.001
Sport frequency at 6 months	3.5						
Never		87 (31)	36 (9)	Ref	Ref	Ref	Ref
Less than once a week		30 (11)	35 (9)	1.84 [1.29;2.62]	<0.001	1.80 [1.22;2.64]	0.003
Once a week		48 (17)	78 (19)	2.12 [1.56; 2.88]	<0.001	2.26 [1.63; 3.13]	<0.001
Twice a week or more		118 (42)	258 (63)	2.34 [1.77; 3.11]	<0.001	2.44 [1.81; 3.30]	<0.001
Hours of sport per week [Mean (SD)]							
Baseline	0	3 (2)	4 (2)	1.08 [1.04; 1.11]	<0.001	1.08 [1.05; 1.12]	<0.001
6 months		3 (2)	4 (2)	1.03 [1.00; 1.06]	0.048	1.10 [1.07; 1.13]	<0.001
**Rehabilitation procedure (N=5)**							
Duration of immobilization (weeks) [Median (IQR)]	0.6	6 (1)	6 (1)	1.00 [0.96; 1.04]	0.931	1.00 [0.96; 1.04]	0.889
Start of passive movements (weeks) [Median (IQR)]	0.8	1 (1)	1 (1)	0.94 [0.89; 0.99]	0.017	0.94 [0.89; 0.99]	0.030
Start of active movements (weeks) [Median (IQR)]	1.2	7 (0)	7 (0)	0.97 [0.94; 1.01]	0.120	0.97 [0.94; 1.01]	0.113
Rehabilitation procedure	0.6						
None		31 (10)	37 (9)	Ref		Ref	
Physiotherapy		218 (73)	318 (77)	1.09 [0.87;1.37]	0.459	1.08 [0.86;1.37]	0.508
Physiotherapy and water therapy		46 (15)	51 (12)	0.97 [0.72; 1.29]	0.816	0.91 [0.67; 1.23]	0.527
Duration of home exercises	1.7						
None		23 (8)	26 (6)	Ref		Ref	
12 weeks or less		24 (8)	40 (10)	1.18 [0.85;1.63]	0.323	1.30 [0.92;1.84]	0.134
More than 12 weeks		244 (84)	346 (84)	1.11 [0.84; 1.45]	0.471	1.17 [0.87; 1.57]	0.471

**Pre- and postoperative scores (N=4)**							
Pain NRS (0-10) [Median (IQR)]							
Baseline	0	6 (3)	5 (3)	0.95 [0.93; 0.98]	<0.001	0.95 [0.92; 0.97]	<0.001
6 weeks	0.7	3 (4)	2 (3)	0.94 [0.92; 0.97]	<0.001	0.95 [0.92; 0.98]	0.001
6 months	2.7	1 (3)	1 (2)	0.90 [0.86; 0.94]	<0.001	0.90 [0.87; 0.94]	<0.001
12 months	5.0	1 (3)	0 (1)	0.85 [0.79; 0.91]	<0.001	0.86 [0.81; 0.92]	<0.001
Constant Murley Score (0-100) [Mean (SD)]							
Baseline	0	49 (18)	53 (17)	1.01 [1.00; 1.01]	0.005	1.01 [1.00; 1.01]	0.005
6 months	5.0	65 (17)	74 (11)	1.02 [1.02; 1.03]	<0.001	1.02 [1.02; 1.03]	<0.001
12 months	7.3	74 (14)	81 (7)	1.03 [1.02; 1.03]	<0.001	1.03 [1.02; 1.03]	<0.001
Abduction strength (kg) [Mean (SD)]							
Baseline	0	3 (3)	3 (3)	1.02 [1.00; 1.04]	0.022	1.02 [1.00; 1.04]	0.038
6 months	5.0	4 (3)	5 (3)	1.06 [1.04; 1.08]	<0.001	1.06 [1.04; 1.08]	<0.001
12 months	5.7	6 (3)	7 (3)	1.05 [1.03; 1.07]	<0.001	1.06 [1.04; 1.08]	<0.001
Shoulder Stiffness Score (0-10) [Median (IQR)]							
Baseline	0	5 (4)	5 (4)	0.98 [0.96; 1.00]	0.117	0.98 [0.95; 1.00]	0.109
6 months	3.1	4 (4)	2 (2)	0.89 [0.86; 0.92]	<0.001	0.89 [0.86; 0.92]	<0.001
12 months	5.7	2 (3)	1 (2)	0.85 [0.81; 0.89]	<0.001	0.85 [0.82; 0.90]	<0.001
**Psychological factors (N=5)**							
PROMIS depression T score (0-100) [Mean (SD)]							
Baseline	0	53 (9)	49 (8)	0.98 [0.97; 0.99]	<0.001	0.98 [0.97; 0.99]	<0.001
6 months	3.5	49 (9)	44 (6)	0.96 [0.95; 0.97]	<0.001	0.96 [0.95; 0.97]	<0.001
12 months	4.3	48 (8)	43 (5)	0.95 [0.94; 0.96]	<0.001	0.95 [0.94; 0.96]	<0.001
PROMIS anxiety T score (0-100) [Mean (SD)]							
Baseline	0	52 (9)	48 (8)	0.98 [0.97; 0.99]	<0.001	0.98 [0.97; 0.99]	<0.001
6 months	3.5	48 (9)	44 (6)	0.96 [0.95; 0.97]	<0.001	0.96 [0.95; 0.97]	<0.001
12 months	4.3	47 (8)	43 (5)	0.96 [0.95; 0.97]	<0.001	0.96 [0.95; 0.97]	<0.001
Motivation at baseline to do sports again after surgery	0	10 (1)	10 (0)	1.29 [1.15; 1.44]	<0.001	1.36 [1.19; 1.55]	<0.001

(0-10) [Median (IQR)]							
Motivation to do sports (0-10) [Mean (SD)]							
12 months	14.8	7 (3)	9 (2)	1.10 [1.06, 1.15]	<0.001	1.12 [1.08, 1.15]	<0.001
24 months	14.5	7 (3)	9 (2)	1.12 [1.08; 1.16]	<0.001	1.19 [1.15; 1.23]	<0.001
Confidence to resume sport at 100% of the initial capacity (0-10) [Median (IQR)]	0	9 (2)	10 (1)	1.13 [1.07; 1.19]	<0.001	1.16 [1.10; 1.23]	<0.001
**Complications (N = 2)**							
Stiffness within 6 months (composite of adverse event and reduced ROM)	0	57 (20)	26 (6)	0.50 [0.36; 0.69]	<0.001	0.60 [0.41; 0.686]	0.006
Ipsilateral Adverse event							
within 12 months	0	118 (39)	84 (20)	0.64 [0.54; 0.77]	<0.001	0.64 [0.54; 0.77]	<0.001
within 24 months	0	131 (44)	93 (22)	0.63 [0.53; 0.75]	<0.001	0.63 [0.53; 0.75]	<0.001

ASA classification = American Society of Anesthesiologists Physical Status classification system; BMI = Body Mass Index; CI = Confidence Interval; IQR = Interquartile Range; ROM = Range of Motion; RR = Risk Ratio; RTS = Return to Sport; SD = Standard Deviation.

Median and IQR are reported for values with Mean/SD <2
