# Supplementary material for: Return to sport after arthroscopic rotator cuff repair: epidemiology and prognostic factors in a Swiss multicentre cohort
Source: Br J Sports Med. 2025 Nov 20;60(2):116–24. doi: 10.1136/bjsports-2025-110358 (PMC12916472; doi:10.1136/bjsports-2025-110358)
Supplement: Supplementary data [file bjsports-60-2-s004.pdf]

eSupplement Table 4a: **Multivariable model of risk factors for RTS**

**ARCR_Pred-RTS final model**	RTS (N = 715)		Complete case		Multiple imputed data	
	No full return (N = 299)	Full return (N = 416)				
**(N = 10)**	N (%)	N (%)	RR (95% CI)	p-value	RR (95% CI)	p-value
**Sociodemographic factors (N = 1)**						
Age at surgery (years) [Mean (SD)]	56 (10)	59 (9)	1.01 [1.00; 1.01]	0.029	1.01 [1.00; 1.01]	0.014
**Surgical and injury-related factors (N = 3)**						
Dominant side operated	226 (76)	285 (69)	0.90 [0.80; 1.02]	0.100	0.92 [0.81; 1.05]	0.205
Traumatic aetiology	144 (48)	245 (59)	1.21 [1.07; 1.37]	0.002	1.21 [1.07; 1.37]	0.003
Operation duration (minutes) [Mean (SD)]	82 (35)	73 (30)	0.99 [0.99; 1.00]	0.011	0.99 [0.99; 1.00]	0.035
**Sport activity (N = 1)**						
Weekly sport hours at 6 months [Mean (SD)]	3 (2)	4 (2)	1.04 [1.01; 1.07]	0.002	1.04 [1.01; 1.07]	0.003
**Pre- and postoperative scores (N = 4)**						
Pain level NRS at baseline (0-10) [Mean (SD)]	6 (2)	5 (2)	0.98 [0.95; 1.00]	0.060	0.97 [0.95; 1.00]	0.017
Shoulder Stiffness Score at 6 months (0-10) [Mean (SD)]	4 (3)	2 (2)	0.96 [0.93; 1.00]	0.033	0.94 [0.90; 0.97]	<0.001
Shoulder Stiffness Score at 12 months (0-10) [Mean (SD)]	2 (2)	1 (1)	0.93 [0.88; 0.98]	0.004	0.97 [0.93; 1.01]	0.127
**Psychological factors (N = 1)**						
Motivation at baseline to return to sports after surgery (0-10) [Mean (SD)]	9 (1)	10 (1)	1.18 [1.05; 1.33]	0.005	1.24 [1.10; 1.41]	0.001
PROMIS depression T score at 12 months (0-100) [Mean (SD)]	47 (8)	43 (5)	0.97 [0.95; 0.98]	<0.001	0.97 [0.95; 0.97]	<0.001

CI = Confidence Interval; PROMIS = Patient Reported Outcomes Measurement Information Systems; RR = Risk Ratio; RTS = Return to Sport; SD = Standard Deviation.

eSupplement Table 4b: **Model performance metrics**

**ARCR_Pred-RTS model performance**	Aikake's Information Criterion	c-index (95% CI)	Pearson goodness-of-fit
**Complete case**	1130.708	0.762 [0.724 to 0.800]	244.082
**Multiple imputed data**	1406.173	0.774 [0.766 to 0.781]	420.173
